# Prevalence and association with environmental factors and establishment of prediction model of atopic dermatitis in pet dogs in China

**DOI:** 10.3389/fvets.2024.1428805

**Published:** 2024-09-25

**Authors:** Yingbo Dong, Long Wang, Kai Zhang, Haibin Zhang, Dawei Guo

**Affiliations:** ^1^MOE Joint International Research Laboratory of Animal Health and Food Safety, College of Veterinary Medicine, Nanjing Agricultural University, Nanjing, China; ^2^Engineering Center of Innovative Veterinary Drugs, Center for Veterinary Drug Research and Evaluation, College of Veterinary Medicine, Nanjing Agricultural University, Nanjing, China; ^3^New Ruipeng Pet Healthcare Group Co., Ltd., Nanjing, China; ^4^Pancreas Center, First Affiliated Hospital of Nanjing Medical University, Nanjing, China

**Keywords:** canine atopic dermatitis, climatic factor, regional factors, model prediction, prevention of disease

## Abstract

Canine atopic dermatitis (CAD) is a common skin disease in dogs. Various pathogenic factors contribute to CAD, with dust mites, environmental pathogens, and other substances being predominant. This research involved comprehensive statistical analysis and prediction of CAD in China, using data from 14 cities. A distributed lag nonlinear model (DLNM) was developed to evaluate the impact of environmental factors on CAD incidence. Additionally, a seasonal auto-regressive moving average (ARIMA) model was used to forecast the monthly number of CAD cases. The findings indicated that CAD mainly occurs during June, July, August, and September in China. There was a positive correlation found between CAD incidence and temperature and humidity, while a negative correlation was observed with CO, PM2.5, and other pollutants.

## 1 Introduction

Canine atopic dermatitis (CAD) is a prevalent clinical syndrome worldwide, rather than a singular disease entity (1). It is increasingly recognized that CAD is a genetically predisposed, progressive, chronically relapsing, inflammatory, and pruritic skin disease with characteristic clinical features associated with immunoglobulin (Ig) E antibodies most commonly directed against environmental allergens (2). What was once simply presented as a type I hypersensitivity to inhaled environmental allergens is now viewed as a multifactorial and complex inflammatory syndrome that may or may not be associated with a demonstrable allergic response, and in which the skin is the main avenue of allergen exposure (3). According to the literature review, dust mites, environmental pathogens, dust, and other substances are the main pathogenic factors of CAD (4). Furthermore, epidemiological surveys showed that the incidence of CAD had a strong relationship with specific regional and climatic factors.

The most common characteristics of CAD are moderate to severe pruritus, often preceded and accompanied by erythema, papules, spontaneous hair loss, shedding, hyperpigmentation, and lichenification (5–7). However, due to the diversity of manifestations, clinical signs are not specific, so a preliminary examination cannot make a definitive diagnosis. This can lead to missed treatment and further skin damage. Moreover, due to the strong genetic tendency and breed specificity, the loss caused by CAD will be more serious (8).

A recent study conducted in Sweden focused on insured dogs, aiming to validate the diagnosis of chronic atopic dermatitis (CAD) in a comprehensive animal insurance database by comparing it with practice records (9). The reported clinical presentations were compared between individuals who were atopic only and dogs with a dual diagnosis of atopy and adverse food reactions. Thus, this comparison offers valuable insights for the early diagnosis of CAD. However, there is no systematic investigation on the pathogenesis of CAD in China.

Recently, the Distributed Lag Non-linear Model (DLNM) is used to assess the delayed impacts of various predictors on an outcome variable. This model effectively captures the intricate, nonlinear relationship between the outcome and its predictors. Based on the comprehensive national case data from XinRuipeng Pet Hospital, the incidence of CAD in dogs, sex, age of onset, clinical manifestations, area of onset, season and duration of onset, and related treatment research progress were demonstrated and predicted, in order to provide valuable insights for clinical diagnosis and treatment, academic research and disease prevention (10).

## 2 Materials and methods

### 2.1 Study population

All the medical data of CAD were obtained from the medical records of Xin Ruipeng hospitals, which include veterinary outpatient clinics throughout China. A diverse range of 14 cities, strategically chosen to represent various regions of the country, were included in the analysis. These 14 cities encompass Central China (Chengdu, Chongqing), North China (Beijing, Tianjin), East China (Shanghai, Nanjing), South China (Shenzhen, Guangzhou), Northwest China (Xi’an, Lanzhou), Southwest China (Changsha, Wuhan), and Northeast China (Shenyang, Changchun). All these data of confirmed dog cases were obtained from January 2021 to December 2022. Sex, age, main complaint, clinic diagnosis, city of treatment, date of hospital visit, and duration of incidence were included in the study (11).

**Figure 1:**
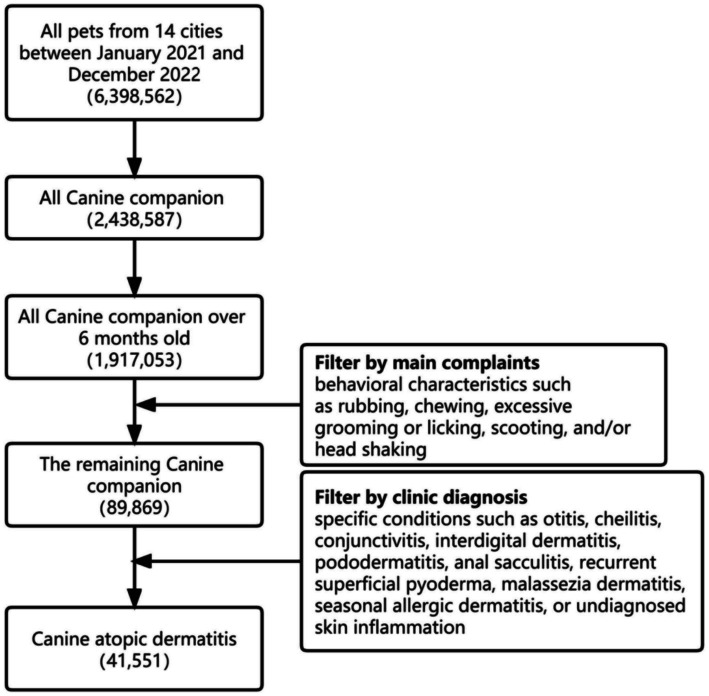
The flowchart for CAD screening.

Since CAD cannot be diagnosed by simple indicators, this study developed a screening process according to the Favrot principles recommended by the International Committee on Animal Allergic Diseases (12). For selection, the process was as follows: Pet type: dog; Pet age: over 6 months old. According to the guidelines for diagnosis and allergen identification of CAD, the main complaints should include behavioral characteristics such as rubbing, chewing, excessive grooming or licking, scooting, and/or head shaking (13). Clinic diagnosis should require specific conditions such as otitis, cheilitis, conjunctivitis, front feet dermatitis, pododermatitis, anal sacculitis, recurrent superficial pyoderma, Malassezia dermatitis, seasonal allergic dermatitis, or undiagnosed skin inflammation. Furthermore, dogs with infectious diseases, virus-related diseases, endocrine-related diseases, immune-mediated diseases, diseases caused by foreign bodies, diseases caused by physiological abnormalities, and tumor-related diseases were excluded based on physical examination, chief complaint information, and laboratory examination in the medical records within the subsequent three-month period. Cases with incomplete medical records and vague physician diagnoses were also excluded. Consequently, a total of 41,551 dog cases were included in the final analysis. The screening process is shown in Figure 1.

### 2.2 Meteorological data

#### 2.2.1 Subjects

The meteorological data and air pollution indicators were obtained from the monitoring and analysis platform^1^ of air quality, specifically the monthly average data of the aforementioned 14 cities, covering the period from 2021 to 2022. This encompassed the monthly average (both maximum and minimum) temperature, average humidity, as well as the monthly average concentrations of PM2.5, PM10, CO, NO_2_, SO_2_, and O_3_ (Supplementary Table S2).

#### 2.2.2 Statistical analysis

In this study, the meteorological data, including temperature, humidity, and air pollutants, were presented as mean with standard deviation (SD). Age was presented as median values with interquartile ranges (IQR). The epidemiological characteristics of CAD, categorized by sex, main complaint, clinic diagnosis, city of treatment, and the year and month of the visit, were presented as number with proportion and were compared by the chi-square test or Fisher’s exact test if necessary. The relationship between the incidence of CAD and meteorological data was analyzed by the Spearman correlation. Furthermore, a Distributed Lag Nonlinear Model (DLNM) was constructed to estimate the effects of ambient temperature, humidity, and air pollutants on CAD using the package “dlnm.” This model is widely used to explore the relations of environmental and health factors. It can effectively capture and evaluate the complex and non-linear relationships between environmental factors and CAD, as well as their delayed effects. The outcome variable was the incidence rate of CAD. The DLNM model was fitted through a cross-basis function simultaneously describing the effect of the monthly mean temperature and its lag structure with maximum lag l on the expected incidence. Each monthly mean ozone concentration of ambient temperature, humidity, and air pollutants were individually input into the model to observe its effects. The seasonal auto-regressive moving average (ARIMA) model was then used to predict the trend of the total number of CAD cases per month using the package “forecast” (14). All these statistical analyses were performed by R version 4.2.2 (R Project for Statistical Computing), and the statistical significance was set at *p* < 0.05.

## 3 Results

### 3.1 Distribution of CAD in 14 cities

The data were collected from 14 cities from January 2021 to December 2022. These datasets encompassed a vast number of 2,438,587 canine cases, with a specific focus on 89,869 instances of pruritus. Out of these, a total of 41,551 dogs were definitively diagnosed with CAD and were subsequently included in the study, accounting for 46.24% of pruritus cases. The median age of these 41,551 cases was 39.0 months, with an interquartile range (IQR) of 19.0–69.0 months, while 23,249 (56.0%) dogs were male (Table 1).

**Table 1:** The prevalence of CAD in China from Jan 2021 to Dec 2022.

	Jan 2021	Feb 2021	Mar 2021	Apr 2021	May 2021	Jun 2021	Jul 2021	Aug 2021	Sep 2021	Oct 2021	Nov 2021	Dec 2021
Total	257,966	212,441	188,293	219,139	283,274	224,457	285,253	312,363	297,321	305,254	278,608	290,028
Canidae	91,239	81,436	77,145	95,761	120,791	91,340	111,584	115,007	108,775	112,520	102,637	104,004
Age (>6 mouth)	70,159	63,248	59,881	75,719	97,005	72,883	89,225	90,280	85,103	88,917	80,973	81,841
Chief complaints screening	2,493	2,114	1967	2,586	3,668	3,809	4,938	5,072	4,614	4,424	3,756	3,430
Diagnostic screening	1,008	815	851	1,131	1,758	1,861	2,463	2,491	2,208	2,054	1,609	1,416

	Jan 2022	Feb 2022	Mar 2022	Apr 2022	May 2022	Jun 2022	Jul 2022	Aug 2022	Sep 2022	Oct 2022	Nov 2022	Dec 2022
Total	279,889	236,710	275,244	248,912	257,037	295,528	309,551	290,268	281,091	301,272	243,618	225,045
Canidae	101,830	87,530	107,321	100,505	102,597	119,033	120,047	109,139	103,852	107,789	87,367	79,338
Age (>6 mouth)	80,729	69,133	83,491	79,057	81,202	96,249	95,813	85,607	80,920	83,363	66,447	59,808
Chief complaints screening	3,090	2,638	3,235	3,249	3,847	5,443	5,716	5,321	4,591	4,275	3,085	2,508
Diagnostic screening	1,218	1,035	1,263	1,321	1,802	2,850	3,034	2,690	2,262	2,010	1,362	1,039

The number of CAD cases in dogs over 6 months old in China were mainly in Beijing (9,673) and Shanghai (7,398), which are the political and economic centers of China, respectively, followed by Shenzhen (4,500) and Nanjing (4,330). Conversely, Changsha (272 cases) and Lanzhou (153 cases), situated in the southwest and northwest regions of China, respectively, exhibit the lowest number of CAD cases. Notably, the cities where CAD prevalence exceeds 2% among dogs older than 6 months include Shanghai, Tianjin, Nanjing, Chengdu, Guangzhou, Shenyang, Shenzhen, and Beijing (Table 2).

**Table 2:** The incidence of CAD in different cities of China.

City	Number of dogs over 6 months old from pet clinics	Number of cases with CAD (Percentage %)	CAD age [months, median (IQR)]	Number of dogs with male CAD (Percentage %)	Site of CAD				
					Ear	Ventral side	Peri-anal region	Eyelid	Plaw
Beijing	477,495	9,673 (2.03)	44.0 (22.0–76.0)	5,743 (59.37)	923 (9.54)	7,175 (74.18)	115 (1.19)	282 (2.92)	1,178 (12.18)
Chengdu	166,352	3,704 (2.23)	35.0 (18.0–61.0)	1,840 (49.68)	263 (7.1)	3,211 (86.69)	25 (0.67)	63 (1.7)	142 (3.83)
Guangzhou	196,671	4,202 (2.14)	44.0 (22.0–74.0)	2,388 (56.83)	333 (7.92)	3,487 (82.98)	41 (0.98)	118 (2.81)	223 (5.31)
Lanzhou	11,271	153 (1.36)	33.0 (16.0–59.0)	73 (47.71)	19 (12.42)	129 (84.31)	1 (0.65)	2 (1.31)	2 (1.31)
Nanjing	192,166	4,330 (2.25)	28.0 (15.0–60.0)	2,414 (55.75)	329 (7.6)	3,665 (84.64)	36 (0.83)	135 (3.12)	165 (3.81)
Shanghai	262,590	7,398 (2.82)	39.0 (19.0–71.0)	4,186 (56.58)	591 (7.99)	6,234 (84.27)	60 (0.81)	203 (2.74)	310 (4.19)
Shenzhen	220,184	4,500 (2.04)	43.0 (21.0–72.0)	2,589 (57.53)	325 (7.22)	3,820 (84.89)	37 (0.82)	100 (2.22)	218 (4.84)
Shenyang	54,768	1,127 (2.06)	32.0 (17.0–55.0)	592 (52.53)	89 (7.9)	918 (81.46)	7 (0.62)	39 (3.46)	74 (6.57)
Tianjin	38,807	907 (2.34)	39.0 (19.0–68.0)	535 (58.99)	69 (7.61)	736 (81.15)	11 (1.21)	28 (3.09)	63 (6.95)
Wuhan	74,283	1,360 (1.83)	40.0 (18.0–69.0)	693 (50.96)	102 (7.5)	1,193 (87.72)	12 (0.88)	28 (2.06)	25 (1.84)
Xi’an	50,207	986 (1.96)	32.0 (16.0–60.0)	516 (52.33)	85 (8.62)	802 (81.34)	8 (0.81)	28 (2.84)	63 (6.39)
Changchun	13,904	272 (1.96)	32.0 (17.0–52.5)	156 (57.35)	21 (7.72)	223 (81.99)	3 (1.1)	18 (6.62)	7 (2.57)
Changsha	65,222	1,255 (1.92)	36.0 (18.0–55.0)	676 (53.86)	95 (7.57)	1,083 (86.29)	7 (0.56)	30 (2.39)	40 (3.19)
Chongqing	93,133	1,684 (1.81)	36.0 (18.0–62.0)	848 (50.36)	92 (5.58)	1,532 (92.96)	6 (0.36)	28 (1.7)	26 (1.58)
Total	1,917,053	41,551 (2.17)	39.0 (19.0–69.0)	23,249 (56.0%)	3,336 (8.03)	34,208 (82.33)	369 (0.89)	1,102 (2.65)	2,536 (6.1)

The leading sites of CAD occurred mainly on the ventral side (34,208, 82.33%). The following high-frequency sites were ears (3,336, 8.03%), paws (2,536, 6.10%), eyelids (1,102, 2.65%), and the peri-anal region (369, 0.89%). These data are summarized in Table 2.

Given that the median age of CAD patients was 39.0 months, we categorized the CAD cases into two groups. The results showed that the CAD cases in 2022, during the fourth quarter of the year, or in South or North China were older (all *p* values were < 0.001). Additionally, a higher proportion of female CAD patients was observed in the first quarter of the year (44.79%), with the largest percentage of female CAD patients reported in Central China (49.81%). However, there was no statistically significant difference in the distribution of CAD cases across different years (Table 3).

**Table 3:** The distribution of CAD in different genders and ages in different regions and seasons.

	Age (Month)				Gender			
	<39.0	≥39.0	*χ* ^2^	*p*-value	Male	Female	*χ* ^2^	*p*-value
Year			82.465	<0.001			1.562	0.211
2021 (%)	10,268 (52.2)	9,401 (47.8)			11,065 (56.79)	8,420 (43.21)		
2022 (%)	10,446 (47.74)	11,436 (52.26)			12,184 (56.17)	9,507 (43.83)		
Period			40.068	<0.001			8.095	0.044
Jan ~ Mar (%)	3,189 (51.53)	3,000 (48.47)			3,389 (55.21)	2,749 (44.79)		
Apr ~ Jun (%)	5,438 (50.71)	5,285 (49.29)			6,095 (57.35)	4,533 (42.65)		
Jul ~ Sep (%)	7,617 (50.28)	7,531 (49.72)			8,497 (56.61)	6,513 (43.39)		
Oct ~ Dec (%)	4,470 (47.1)	5,021 (52.9)			5,268 (56.04)	4,132 (43.96)		
Region of China			420.98	<0.001			158.24	<0.001
Central (%)	2,953 (54.81)	2,435 (45.19)			2,688 (50.19)	2,668 (49.81)		
East (%)	6,303 (53.74)	5,425 (46.26)			6,600 (57.27)	4,925 (42.73)		
North (%)	4,776 (45.14)	5,804 (54.86)			6,278 (59.58)	4,259 (40.42)		
Northeast (%)	818 (58.47)	581 (41.53)			748 (53.66)	646 (46.34)		
Northwest (%)	670 (58.82)	469 (41.18)			589 (52.40)	535 (47.60)		
South (%)	3,829 (44.00)	4,873 (56.00)			4,977 (57.47)	3,683 (42.53)		

### 3.2 Relationship between CAD and environment

From January 2021 to December 2022, the median monthly maximum temperature, minimum temperature and humidity were 22°C, 13°C and 70%, respectively. The median monthly concentrations of PM2.5, PM10, CO, NO_2_, SO_2_, and O_3_ were 27 (IQR: 20, 32) μg/m^3^, 51 (IQR: 38, 54) μg/m^3^, 0.672 (IQR: 0.589, 0.707) mg/m^3^, 30 (IQR: 23, 31) μg/m^3^, 7 (IQR: 5, 8) μg/m^3^, and 92.0 (IQR: 66.8, 92.2) μg/m^3^, respectively (Supplementary Table S2).

**Figure 2:**
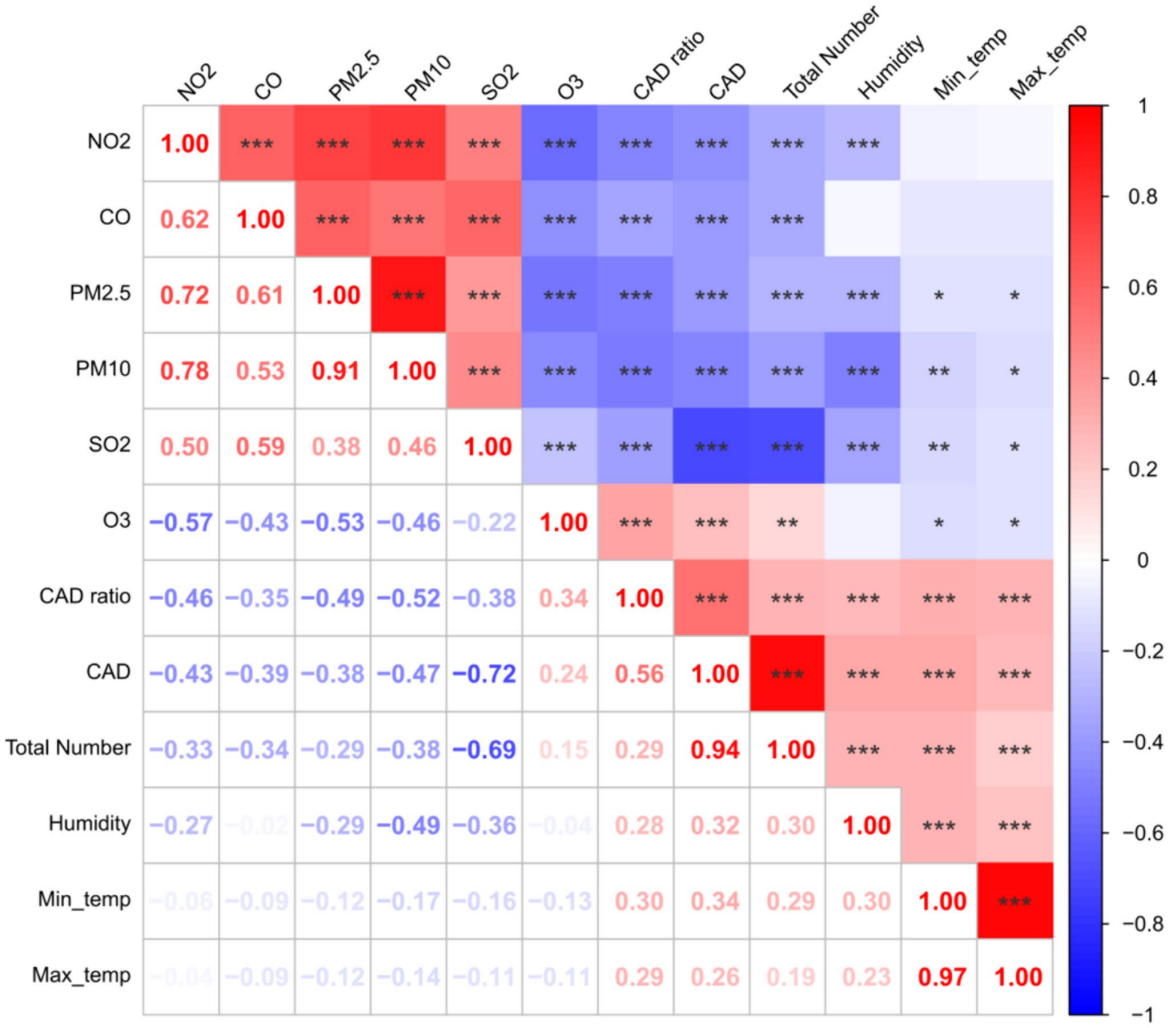
The correlations between temperature, humidity and air pollutants, and the incidence of CAD cases. The lower left part represented the *R* value of spearman correlation; In the upper right part, red signifies a positive correlation, while blue represents a negative correlation. The intensity of the color corresponds to the magnitude of the correlation. Asterisks (*, **, ***) indicate statistical significance at the 0.05, 0.01, and 0.0001 levels, respectively.

The results of Spearman correlation analysis between temperature, humidity, and air pollutants and the incidence of CAD cases are presented in Figure 2. The incidence of CAD is positively correlated with temperature, humidity, and O_3_, while it is negatively correlated with the concentration of PM2.5, PM10, CO, NO_2_, and SO_2_, and all these correlations are statistically significant (*p* < 0.0001). The correlation between air pollutants, temperature, and the number of CAD cases in each city was shown in Supplementary Table S1.

According to Table 4, there was a relationship between the incidence of CAD and temperature, humidity, and air pollutants estimated by the DLMN model. Nine environmental factors were incorporated into the single-pollution model to analyze the delayed impact on incidence of CAD. Statistically significant associations were observed between the incidence of CAD and maximum temperature, minimum temperature, humidity and the concentrations of PM2.5, PM10, CO, NO_2_, SO_2_, and O_3_. The peak effects occurred with lags of 1, 1, 1, 1, 1, 0, 1, 1, and 1 month, respectively. The coefficients for the increase in these 9 Nine environmental factors were determined to be 0.78 (0.45 ~ 1.11), 0.68 (0.42 ~ 0.94), 0.47 (0.22 ~ 0.71), −0.90 (−1.14 ~ −0.66), −1.10 (−1.3 ~ −0.89), 0.54 (0.11 ~ 0.97), −0.81 (−1.01 ~ −0.62), −0.52 (−0.74 ~ −0.30), and 0.55 (0.38 ~ 0.72), respectively. The effects of maximum temperature, minimum temperature, and humidity on the incidence of CAD were all positive and statistically significant. On the other hand, the effects of air pollutants PM2.5, PM10, NO_2_, and SO_2_ on the incidence of CAD were all negative and statistically significant. However, the effects of CO and O_3_ on the incidence of CAD were both positive.

**Table 4:** Effects of different climatic factors on the incidence of CAD.

	Lag0	Lag1	Lag2
Maximum temperature	0.62 (0.09 ~ 1.15)*	0.78 (0.45 ~ 1.11)***	0.03 (−0.52 ~ 0.59)
Minimum temperature	0.70 (0.23 ~ 1.17)**	0.68 (0.42 ~ 0.94)***	0.25 (−0.26 ~ 0.76)
Humidity (%)	−0.08 (−0.54 ~ 0.37)	0.47 (0.22 ~ 0.71)***	0.04 (−0.46 ~ 0.54)
PM2.5 (μg/m^3^)	0.17 (−0.12 ~ 0.45)	−0.90 (−1.14 ~ −0.66)***	0.10 (−0.50 ~ 0.71)
PM10 (μg/m^3^)	0.04 (−0.22 ~ 0.30)	−1.10 (−1.3 ~ −0.89)***	0.12 (−0.37 ~ 0.61)
CO (mg/m^3^)	0.54 (0.11 ~ 0.97)*	−0.30 (−0.62 ~ 0.02)	−0.22 (−0.88 ~ 0.44)
NO_2_ (μg/m^3^)	0.06 (−0.23 ~ 0.36)	−0.81 (−1.01 ~ −0.62)***	−0.19 (−0.64 ~ 0.27)
SO_2_ (μg/m^3^)	0.13 (−0.09 ~ 0.35)	−0.52 (−0.74 ~ −0.30)***	−0.36 (−0.81 ~ 0.09)
O_3_ (μg/m^3^)	−0.31 (−0.61 ~ −0.01)*	0.55 (0.38 ~ 0.72)***	0.19 (−0.20 ~ 0.58)

*Represented as *p* < 0.05, **represented as *p* < 0.01, ***represented as *p* < 0.0001.

### 3.3 Prediction of CAD in different cities

**Figure 3:**
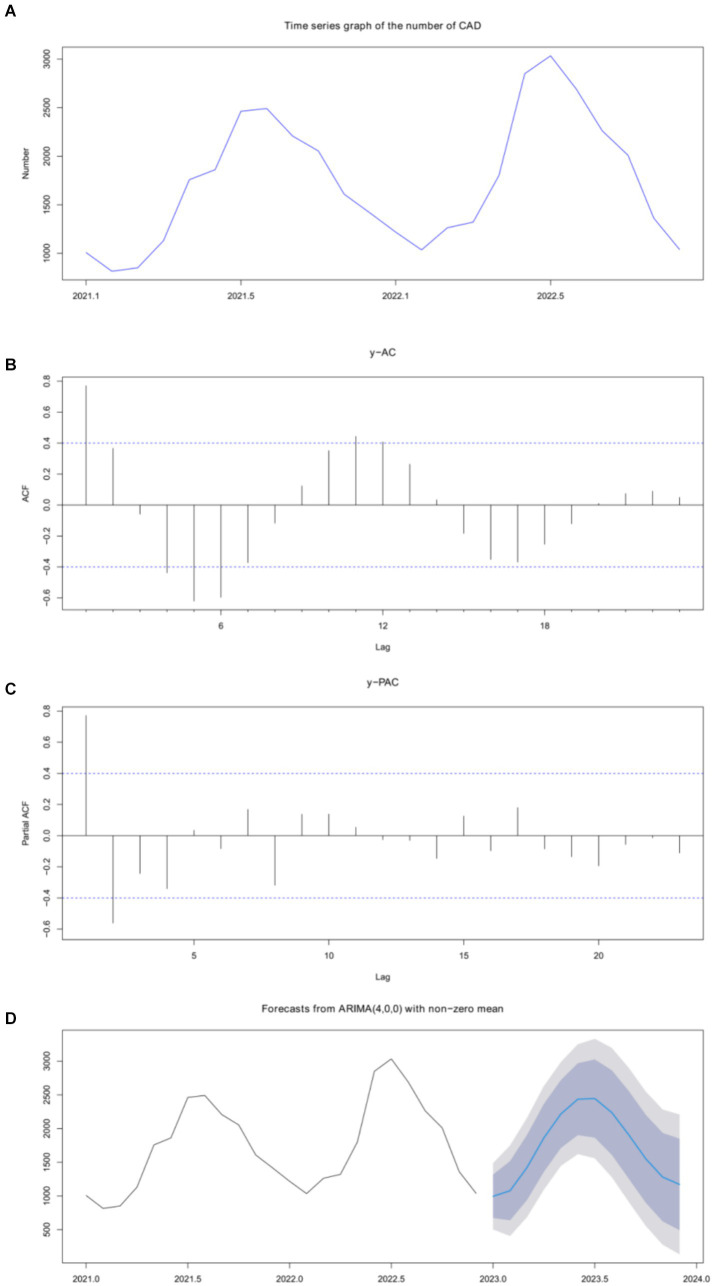
Time series was used to analyze and predict the incidence of CAD. **(A)** CAD quantity outcomes were predicted using time series. **(B)** Analysis of the original sequence. **(C)** Differential sequence analysis. **(D)** SARIMA (4,0,0) model.

We used the time series method to analyze the number of CAD cases per month. The time series plot is shown in Figure 3A. As the *p* value was 0.072 in the Augmented Dickey-Fuller test, the seasonal ARIMA model was used to build the prediction model. Referring to the autocorrelation function (ACF) and partial autocorrelation function (PACF) figures (Figures 3B,C) of the original series and difference series, the SARIMA model was fitted using the auto-arima function, which automatically selects the model’s parameters and returns the SARIMA model with the minimum AIC value. The SARIMA (4,0,0) model was finally constructed (Figure 3D) with model AICc = 348.5. The result of the Kolmogorov–Smirnov nonparametric test showed that *D* = 0.142 and *p* = 0.670 with the normally distributed residuals of the model. The predicted number of cases in 2023 is shown in Table 5.

**Table 5:** Estimated number of CAD cases in 2023.

Month	Estimated number	95%CI of estimated number
Jan	995	504–1,487
Feb	1,077	406–1,748
Mar	1,422	686–3,258
Apr	1,863	1,095–2,632
May	2,217	1,447–2,986
Jun	2,436	1,622–3,251
Jul	2,445	1,559–3,332
Aug	2,238	1,280–3,196
Sep	1,908	915–2,901
Oct	1,554	558–2,550
Nov	1,280	278–2,283
Dec	1,171	135–2,207

## 4 Discussion

In this study, a comprehensive survey was conducted across pet stores in 14 cities in China to gain insights into the incidence of Canine Atopic Dermatitis (CAD) among pet dogs in the country. Additionally, the DLNM model was employed to investigate the influence of nine environmental factors, including temperature, humidity, and air pollutants, on the incidence of CAD. Furthermore, the SARIMA model was utilized to anticipate the future occurrence of CAD in pet dogs. These findings aim to offer valuable insights to subsequent pet medical institutions.

### 4.1 The influence of air pollutants on the incidence of CAD

#### 4.1.1 Effect of PM2.5 on the incidence of CAD

PM2.5, which refers to fine particulate matter with a diameter of 2.5 microns or less, is an air pollutant (15). When PM2.5 levels rise, it usually has a negative impact on human health, such as respiratory diseases, cardiovascular diseases, and even cancer. However, the above data suggest that for dogs, elevated PM2.5 levels may actually reduce the incidence of skin diseases such as CAD.

We believe that the reason for this difference may be that when the content of PM2.5 in the air increases, the water vapor content in the air will relatively decrease, making the air drier. A dry environment can breed certain canine skin diseases, thus reducing the incidence of CAD. In addition, there is another hypothesis for this phenomenon. Literature has shown that when the content of air pollutants increases, people’s outdoor activities will decrease (16). Therefore, it can be speculated that when the content of PM2.5 increases, people’s outdoor activities will decrease, which will lead to the reduction of outdoor activities of pet dogs, thus reducing the skin exposure to air pollutants such as PM2.5. As a result, the incidence of CAD is reduced. At the same time, some studies have shown that the increase of air pollutants such as PM2.5 will also make the animal body surface drier, thereby reducing the infection of some pathogens (17). However, some studies have also shown that PM2.5 will increase the incidence of CAD in dogs and humans alike, and the study also mentioned that the PM2.5 content in the homes of dogs with CAD was higher than that of dogs without CAD (15). We think this may be related to the different dog-raising habits in different regions and the different attitudes of pet owners toward air pollution. The specific mechanism of PM2.5’s effect on CAD requires further research.

#### 4.1.2 Effect of O_3_ on the incidence of CAD

In general, elevated ozone concentrations have a negative impact on dog skin health and may lead to an increased incidence of canine dermatitis for the following reasons: O_3_ damages skin tissues (18): High levels of O_3_ can damage a dog’s skin and reduce its immunity, which increases the incidence of dermatitis. O_3_ stimulates inflammation: O_3_ can cause inflammation, which can increase the incidence of existing skin diseases. High temperature and humidity make skin more vulnerable: In high temperature and humidity, O_3_ molecules can irritate skin cells and damage the integrity of skin cell membranes, making skin more vulnerable to damage and irritation (19–21). In conclusion, although O_3_ is a gas that is harmful to the health of dogs, its association with skin diseases needs to be further studied and explored as its effects may be influenced by many other factors.

### 4.2 The impact of geographical location of living environment on the incidence of CAD

**Figure 4:**
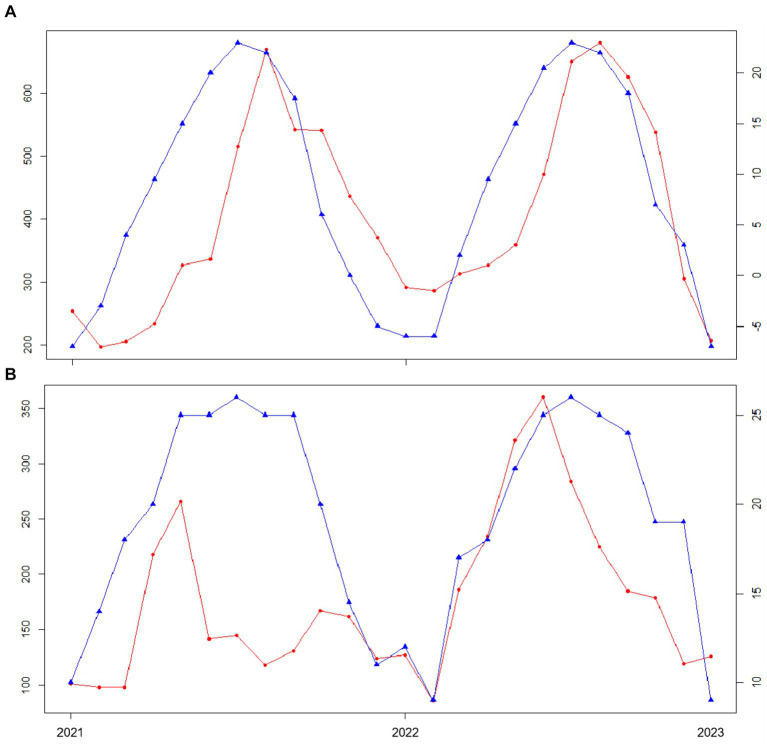
Plot of CAD incidence versus temperature in different cities. The red line represents the change of CAD incidence, and the blue line represents the change of temperature. **(A)** Graph of the relationship between Beijing temperature and the number of CAD cases. **(B)** Graph of the relationship between Guangzhou temperature and the number of CAD cases.

The differences in the geographical environment of the dogs are caused by the different geographical locations in China. As can be seen from Figure 4, the incidence of CAD varies significantly with time and temperature in different regions. Taking Beijing and Guangzhou as an example, it can be seen from the figure that the temperature change trend of Beijing and Guangzhou is similar, but the time curves of CAD incidence are quite different. There was a one-month lag between the CAD incidence curve and the temperature curve in Beijing, but not in Guangzhou Therefore, it can be seen that the incidence of CAD is indeed affected by geographical location factors.

In addition, due to the differences in economic development in different cities, the treatments of pet owners after the onset of the disease, the total number of pet dogs, and the distribution of pet hospitals will have an impact on the incidence of CAD shown in the data (22). Therefore, it can be speculated that the impact of geographical location on the incidence of CAD is a complex process, and its influencing factors are also diverse. However, through this survey on the number of CAD cases, the trend of the incidence of CAD in a specific city with time can be preliminarily obtained, so as to effectively infer the incidence of CAD in the future (23).

### 4.3 Influence of canine gender on CAD

In this survey, we found that the incidence of CAD in male dogs is higher than that in female dogs nationwide, and this situation has not changed over time. Therefore, although there is no clear evidence that the incidence of CAD is directly related to gender, we can infer that the incidence of CAD in male dogs is slightly higher than that in female dogs. This is similar to the results of a Hungarian survey of canine atopic dermatitis (23). However, in another report earlier (24), it was also inferred that CAD is more common in females than in males, so this investigation can only prove that the incidence of CAD in males is higher than that in females in the treated dogs in the above areas, and the specific reason needs to be proved by further experiments.

### 4.4 Main onset time of CAD in China

In addition, as shown in Figure 2, the incidence of CAD peaks in June, July, August, and September, which is also a high-risk period for parasitic infections, fungal infections, and other skin diseases, as shown by other studies (25). Therefore, pet dogs should strengthen cleaning measures during this period, reduce exposure to air pollutants, keep their body surface clean and dry, and undergo regular checkups, so as to reduce the incidence of CAD and other skin diseases.

## 5 Conclusion

Based on the case data compiled by Xin Ruipeng Hospitals, CAD predominantly manifests during June, July, August, and September in China. Temperature and humidity were positively correlated with the incidence of CAD, while most of these common air pollutants, such as PM2.5, PM10, NO_2_, and SO_2_, were negatively correlated. The effects of these environmental factors except CO were delayed for about 1 month. However, relevant studies have shown that exposure to PM2.5 in animals can cause skin barrier damage and lead to the development and deterioration of CAD (26–29), which is contrary to the results obtained from the survey data. Therefore, it is speculated that there are other social and environmental factors affecting the results in China. It is imperative for pet dogs to enhance hygiene measures during these months, minimize exposure to atmospheric pollutants, maintain cleanliness and dryness of their coats, and undergo periodic health screenings. By adhering to these preventive measures, the likelihood of CAD and other skin disorders can be reduced.

## Data Availability

The raw data supporting the conclusions of this article will be made available by the authors, without undue reservation.

## Footnotes

^1^ http://tianqihoubao.com/
