# Supplementary material for: Prevalence and association with environmental factors and establishment of prediction model of atopic dermatitis in pet dogs in China
Source: Front Vet Sci. 2024 Sep 25;11:1428805. doi: 10.3389/fvets.2024.1428805 (PMC11461458; doi:10.3389/fvets.2024.1428805)
Supplement: Supplementary file 1 [file Data_Sheet_1.zip › Supplementary Material Presentation/Fig 2.pdf]

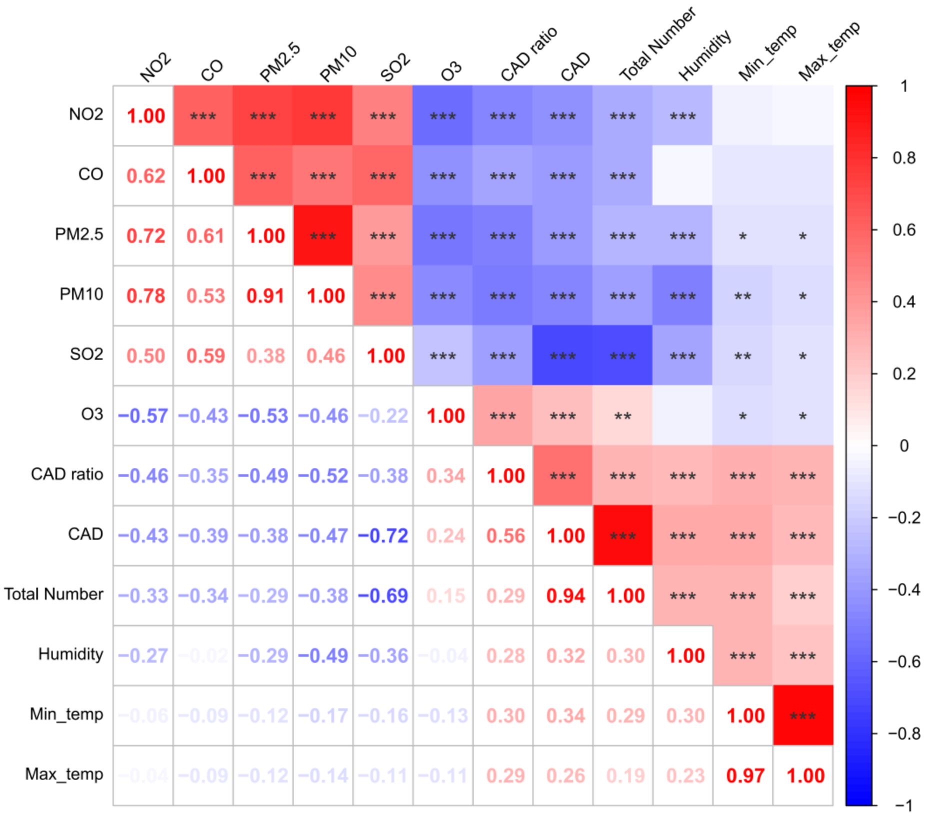

Fig 2. The correlations between temperature, humidity and air pollutants, and the incidence of CAD cases. The lower left part represented the R value of spearman correlation;In the upper right part, red signifies a positive correlation, while blue represents a negative correlation. The intensity of the color corresponds to the magnitude of the correlation. Asterisks (*, **, ***) indicate statistical significance at the 0.05, 0.01, and 0.0001 levels, respectively.
