# Supplementary material for: Prevalence and association with environmental factors and establishment of prediction model of atopic dermatitis in pet dogs in China
Source: Front Vet Sci. 2024 Sep 25;11:1428805. doi: 10.3389/fvets.2024.1428805 (PMC11461458; doi:10.3389/fvets.2024.1428805)
Supplement: Supplementary file 1 [file Data_Sheet_1.zip › Supplementary Material Presentation/Fig3.pdf]

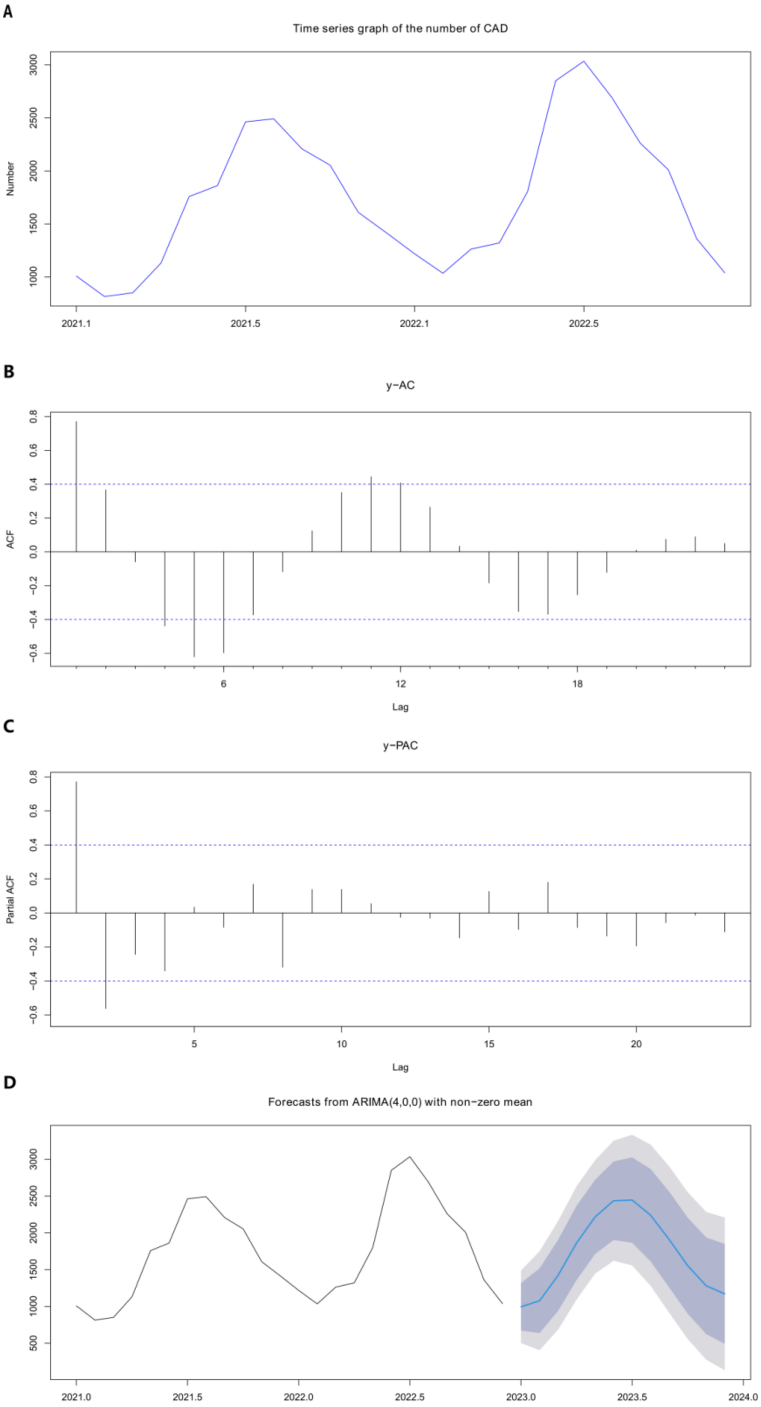

Fig. 3 Time series was used to analyze and predict the incidence of CAD.(A) CAD quantity outcomes were predicted using time series. (B) Analysis of the original sequence. (C) Differential sequence analysi .(D) SARIMA (4,0,0) Model.
