# Supplementary material for: Prevalence and association with environmental factors and establishment of prediction model of atopic dermatitis in pet dogs in China
Source: Front Vet Sci. 2024 Sep 25;11:1428805. doi: 10.3389/fvets.2024.1428805 (PMC11461458; doi:10.3389/fvets.2024.1428805)
Supplement: Supplementary file 1 [file Data_Sheet_1.zip › Supplementary Material Presentation/Fig4.pdf]

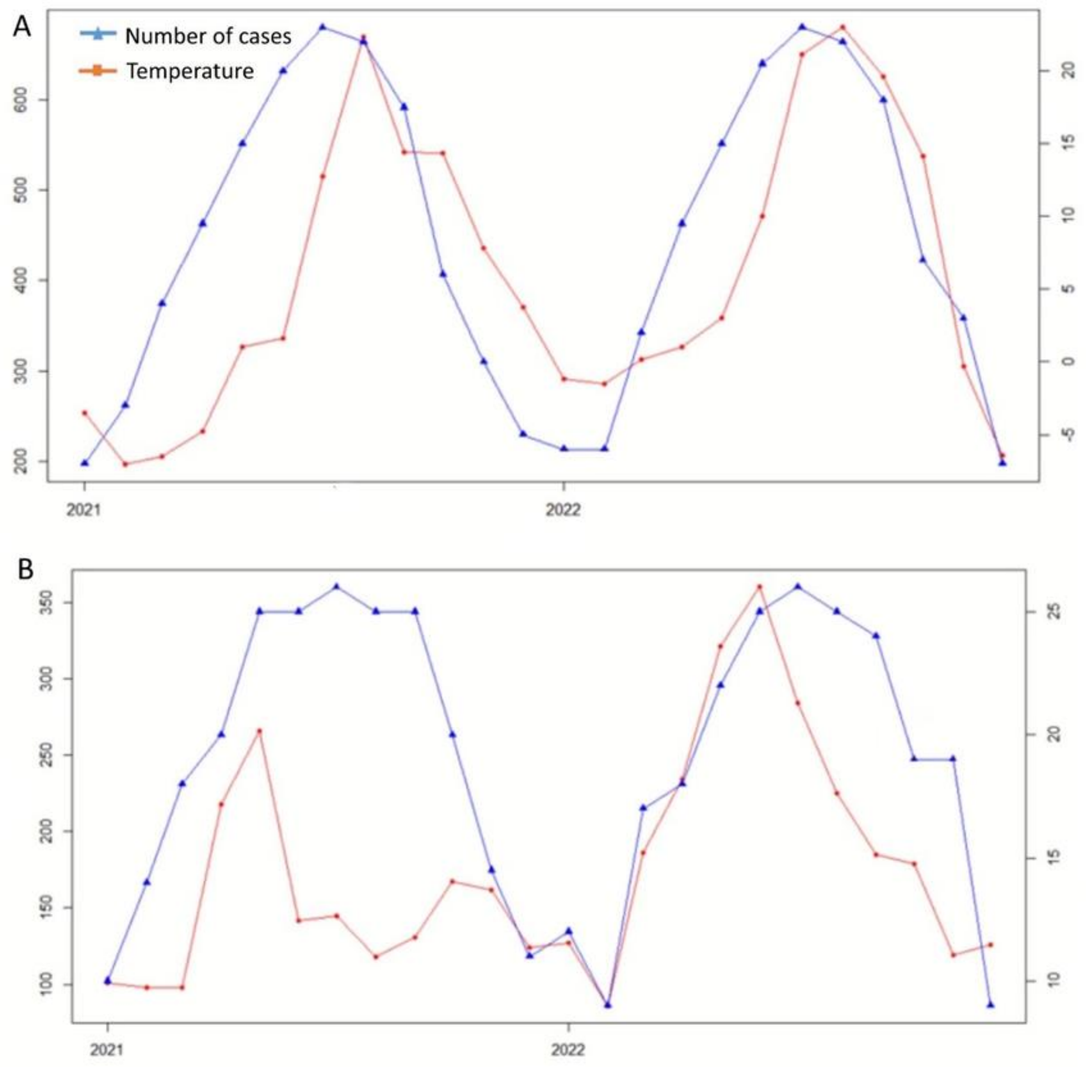

Fig.4 Plot of CAD incidence versus temperature in different cities. (A)Graph of the relationship between Beijing temperature and the number of CAD cases. (B) Graph of the relationship between Guangzhou temperature and the number of CAD cases.
