# Supplementary material for: Prevalence and association with environmental factors and establishment of prediction model of atopic dermatitis in pet dogs in China
Source: Front Vet Sci. 2024 Sep 25;11:1428805. doi: 10.3389/fvets.2024.1428805 (PMC11461458; doi:10.3389/fvets.2024.1428805)
Supplement: Supplementary file 1 [file Data_Sheet_1.zip › Supplementary Material Presentation/Supplementary_Material.docx]

Supplementary Material

Table S1 Basic information on temperature, humidity and air pollutants in 14 Chinese cities

| Spearman R  P | Number of cases | proportion | humidity | max_temp | min_temp | PM2.5 | PM10 | CO | NO_2_ | SO_2_ | O_3_ |
| --- | --- | --- | --- | --- | --- | --- | --- | --- | --- | --- | --- |
| Number of cases |  | 0.51 | 0.19 | 0.2 | 0.23 | -0.34 | -0.37 | -0.4 | -0.39 | -0.53 | 0.22 |
| Proportion | <0.001 |  | 0.32 | 0.27 | 0.28 | -0.42 | -0.45 | -0.31 | -0.42 | -0.33 | 0.34 |
| Humidity | 0.0006 | <0.001 |  | 0.32 | 0.39 | -0.19 | -0.43 | -0.1 | -0.27 | -0.39 | -0.03 |
| Max_temp | 0.0002 | <0.001 | <0.001 |  | 0.97 | -0.16 | -0.15 | -0.16 | -0.05 | -0.2 | -0.08 |
| Min_temp | <0.001 | <0.001 | <0.001 | <0.001 |  | -0.15 | -0.18 | -0.17 | -0.07 | -0.27 | -0.1 |
| PM2.5 | <0.001 | <0.001 | 0.0005 | 0.0031 | 0.007 |  | 0.88 | 0.62 | 0.67 | 0.35 | -0.56 |
| PM10 | <0.001 | <0.001 | <0.001 | 0.0047 | 0.0011 | <0.001 |  | 0.61 | 0.77 | 0.49 | -0.47 |
| CO | <0.001 | <0.001 | 0.0611 | 0.0033 | 0.0013 | <0.001 | <0.001 |  | 0.69 | 0.69 | -0.43 |
| NO_2_ | <0.001 | <0.001 | <0.001 | 0.3466 | 0.2082 | <0.001 | <0.001 | <0.001 |  | 0.51 | -0.56 |
| SO_2_ | <0.001 | <0.001 | <0.001 | 0.0002 | <0.001 | <0.001 | <0.001 | <0.001 | <0.001 |  | -0.28 |
| O_3_ | <0.001 | <0.001 | 0.5476 | 0.1678 | 0.0779 | <0.001 | <0.001 | <0.001 | <0.001 | <0.001 |  |

Table S2 Climate data of 14 cities in China in 2021-2022

|  | Humidity | Max temp | Min temp | PM2.5  （μg/m3） | PM10  （μg/m3） | CO（mg/m3） | NO2 （μg/m3） | SO2  （μg/m3） | O3  （μg/m3） |
| --- | --- | --- | --- | --- | --- | --- | --- | --- | --- |
| Min. | 28 | -12 | -20 | 7 | 18 | 0.33 | 11 | 2 | 33 |
| 1st | 58 | 14 | 5 | 20 | 38 | 0.59 | 23 | 5 | 66.75 |
| Median | 70 | 22 | 13 | 27 | 51 | 0.67 | 30 | 7 | 92 |
| 3rd | 78 | 30 | 21 | 40 | 65 | 0.80 | 37 | 9 | 116 |
| Max. | 93 | 41 | 33 | 110 | 152 | 1.74 | 72 | 28 | 174 |
| Mean | 66.82 | 20.94 | 12.03 | 31.97 | 54.26 | 0.71 | 31.48 | 7.94 | 92.21 |
| sd | 13.46 | 10.33 | 10.79 | 16.51 | 22.23 | 0.18 | 10.90 | 4.15 | 32.18 |
