# Supplementary figures and images for: Prevalence and association with environmental factors and establishment of prediction model of atopic dermatitis in pet dogs in China
Source: Front Vet Sci. 2024 Sep 25;11:1428805. doi: 10.3389/fvets.2024.1428805 (PMC11461458; doi:10.3389/fvets.2024.1428805)

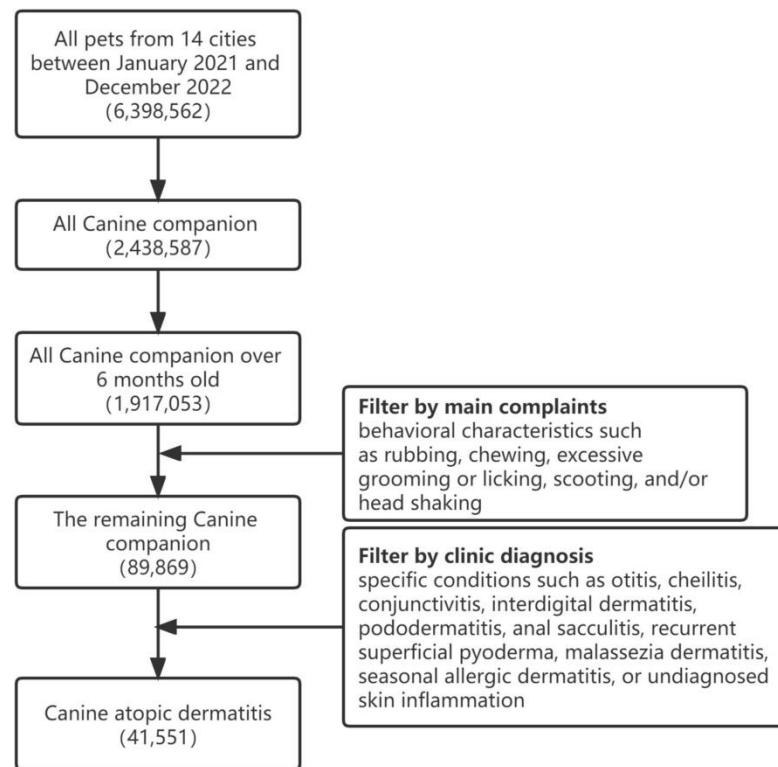

Fig 1 The Flowchart for CAD Screening

Supplement: Supplementary file 1 [file Data_Sheet_1.zip › Supplementary Material Presentation/Fig 1.pdf]
